# Plant lighting system with five wavelength-band light-emitting diodes providing photon flux density and mixing ratio control

**DOI:** 10.1186/1746-4811-8-46

**Published:** 2012-11-22

**Authors:** Akira Yano, Kazuhiro Fujiwara

**Affiliations:** 1 Faculty of Life and Environmental Science, Shimane University, 1060 Nishikawatsu, Matsue, Shimane, 690-8504, Japan; 2 Graduate School of Agricultural and Life Sciences, The University of Tokyo, 1-1-1 Yayoi, Bunkyo, Tokyo, 113-8657, Japan

**Keywords:** LED, Spectrum, Spectral photon flux density, Spectral irradiance, Phototropism, Oat coleoptiles, Red light, Blue light, Gradient light

## Abstract

**Background:**

Plant growth and development depend on the availability of light. Lighting systems therefore play crucial roles in plant studies. Recent advancements of light-emitting diode (LED) technologies provide abundant opportunities to study various plant light responses. The LED merits include solidity, longevity, small element volume, radiant flux controllability, and monochromaticity. To apply these merits in plant light response studies, a lighting system must provide precisely controlled light spectra that are useful for inducing various plant responses.

**Results:**

We have developed a plant lighting system that irradiated a 0.18 m^2^ area with a highly uniform distribution of photon flux density (PFD). The average photosynthetic PFD (PPFD) in the irradiated area was 438 micro-mol m^–2^ s^–1^ (coefficient of variation 9.6%), which is appropriate for growing leafy vegetables. The irradiated light includes violet, blue, orange-red, red, and far-red wavelength bands created by LEDs of five types. The PFD and mixing ratio of the five wavelength-band lights are controllable using a computer and drive circuits. The phototropic response of oat coleoptiles was investigated to evaluate plant sensitivity to the light control quality of the lighting system. Oat coleoptiles irradiated for 23 h with a uniformly distributed spectral PFD (SPFD) of 1 micro-mol m^–2^ s^–1^ nm^–1^ at every peak wavelength (405, 460, 630, 660, and 735 nm) grew almost straight upwards. When they were irradiated with an SPFD gradient of blue light (460 nm peak wavelength), the coleoptiles showed a phototropic curvature in the direction of the greater SPFD of blue light. The greater SPFD gradient induced the greater curvature of coleoptiles. The relation between the phototropic curvature (deg) and the blue-light SPFD gradient (micro-mol m^–2^ s^–1^ nm^–1^ m^–1^) was 2 deg per 1 micro-mol m^–2^ s^–1^ nm^–1^ m^–1^.

**Conclusions:**

The plant lighting system, with a computer with a graphical user interface program, can control the PFD and mixing ratios of five wavelength-band lights. A highly uniform PFD distribution was achieved, although an intentionally distorted PFD gradient was also created. Phototropic responses of oat coleoptiles to the blue light gradient demonstrated the merit of fine controllability of this plant lighting system.

## Background

Light-emitting diodes (LEDs) offer many benefits for applications in plant studies and potentially in commercial cultivation. Their solidity and longevity enable easier installation and manipulation compared to conventional lighting devices such as incandescent and fluorescent lamps, which have fragile glass sheaths [1-3]. Their mechanical reliability makes LED light sources movable [4], even with some speed and vibration, above plant canopies. Another feature of LEDs is that their chip volume is generally much smaller than that of whole plants. This beneficial feature enables manifold designs of light sources from irradiation inside a single tissue culture vessel, using only a few LEDs [5] to irradiation of greenhouse crops using large LED arrays [6] according to the cultivation scale. The small volume of LEDs also widens the variety of available plant irradiation methods such as irradiation of specific organs [7] and unrestricted directional irradiation [6,8]. Close proximity irradiation [8] is also possible using visible-spectrum LEDs that do not emit collateral infrared radiation, which would result in potentially undesirable increase in plant temperature. Radiant flux is controllable by regulating the electric power input to LEDs. Because of this merit, excessive electricity dissipation can be avoided and desirable plant responses can be derived by feeding minimal electric power to LEDs. An LED dim lighting for seedling storage [9] is a demonstration of plant quality improvement using minimal electric power input. Dynamic control of lighting for beneficial cultivation can be realized by regulating the LED input power temporally in response to plant condition feedback [9]. Not only is day–night periodic irradiation control possible; high-frequency on-off cycling can also be done using LEDs. Thereby, rapidly occurring photochemical reactions can be investigated [10]. At 50% peak wavelength the band of light emitted from an LED is generally narrow, except for white LEDs with a fluorescent material, which enables users to select a specific light wavelength range or combinations of ranges using various LEDs. Moreover, LED lighting with some plant-photoreceptor-activating wavelengths in addition to necessary background light is anticipated for modification of specific plant functions [6]. Optimum spectrum lighting is also desirable for efficient energy usage of plant lighting [11].

Room exists for engineering efforts to improve LED characteristics. The conversion efficiency for electric energy into light energy is reported as around 20%–30% [1,12]. The remaining input electrical energy is transformed into heat. That heat must be removed from LEDs to avoid damage to LED chips and to provide stable light emissions [3,11,12]. Another challenge is to reduce the initial LED cost, which is still higher than that of fluorescent lamps, although it is decreasing rapidly [3,12]. Notwithstanding these hurdles, the numerous and important merits of LEDs described above underpin their new lighting value for plant researchers and plant growers. To apply these merits in plant studies and cultivation, light spectra that are valid for inducing various plant responses should be clarified precisely and exhaustively. For this reason, a multi-peak-wavelength plant lighting system that irradiates a wide area with a highly uniform distribution of photon flux density (PFD) is necessary. Such a plant lighting system is expected to provide an appropriate photosynthetic PFD (PPFD; wavelengths of 400–700 nm) for growing numerous seedlings at once and for cultivating some mature leafy vegetables. Such systems are also expected to provide controllability of PFDs and of the mixing ratio of the respective light spectra emitted from all LED types used in the lighting system.

We developed a plant lighting system that irradiates light including violet, blue, orange-red, red, and far-red wavelength bands using five LED types. The lighting system was designed for indoor applications where no sunlight is available. For this reason, blue, orange-red, and red light were necessary for driving plant photosynthesis. In addition, violet and far-red types of LEDs were included in the lighting system to extend its usability for applications such as secondary metabolite synthesis and photomorphogenesis studies. These wavelength bands are known to be important independently or complementarily for plant photosynthesis, pigment synthesis, growth, and development [13-22]. The lighting system can produce high PPFD sufficient for growing vegetables from seedlings to mature plants. The irradiated area of 30 cm × 60 cm is suitable for growing many seedlings concurrently using a conventional cell tray. Furthermore, the mixing ratio of PFDs of five wavelength bands and the distribution of PFDs are controllable using a computer and drive circuits to extend the usefulness of this system for diverse plant studies. The phototropic responses of oat coleoptiles [23-28] induced using a blue light gradient demonstrated the fine lighting controllability of this developed plant lighting system.

## Methods

### LEDs

An LED panel (40 cm × 70 cm) was fabricated using 2800 indicator-type LEDs of 3-mm diameter. The LEDs are of five types: violet (L405R-36; Epitex Inc., Kyoto, Japan), blue (L460-36; Epitex Inc., Kyoto, Japan), orange-red (L630-36; Epitex Inc., Kyoto, Japan), red (SRK3-3A80-LE; Toricon, Shimane, Japan), and far-red (L735-36 AU; Epitex Inc., Kyoto, Japan), each of which emits a specific peak wavelength (*λ*_p_) light (Table 1). The *λ*_p_ of the violet LED was at 405 nm, which provides the shortest wavelength light that is effective for photosynthesis [29,30]. Its wavelength range covers a part of ultraviolet-A, which affects the synthesis of secondary metabolites in some plant species [31]. The blue-type LED emits light with 460 nm *λ*_p_, which activates photosynthesis [22,29,30,32] and which induces phototropin-mediated and cryptochrome-mediated plant responses [33-38]. Blue light also enhances the accumulation of pigments such as chlorophylls in cucumber leaves [22], carotenoids in citrus juice sacs cultured *in vitro*[39], and anthocyanins in lettuce leaves [21]. Orange-red light with 630 nm *λ*_p_ enhances photosynthesis [29,30]. Enhancement of antioxidant activities in pea seedlings at *λ*_p_ = 630 nm has also been reported [19]. The wavelengths of 660–735 nm cover the action spectra of plant responses such as flowering [40,41], morphogenesis [36,40,42-44], and photoperiodic responses through red/far-red-light receptor phytochromes [40,45]. The wavelength range around 660 nm also occupies a primary part of the photosynthetically active radiation [7,29,30,46].

**Table 1:** Description of LEDs used for the LED panel

**LED type**	**Model code**	***λ***_p_^*1^**(nm)**	***V***_Fs_^*2^**(V)**	***I***_Fs_^*3^**(mA)**	N^*4^	NSC^*5^	NPC^*6^
violet	L405R-36^*7^	405	3.5	20	448	16	4
blue	L460-36^*7^	460	3.2	20	784	14	8
orange-red	L630-36^*7^	630	2.1	20	672	24	4
red	SRK3-3A80-LE^*8^	660	2.1	20	448	16	4
far-red	L735-36 AU^*7^	735	1.8	50	448	16	4

*1 Peak wavelength; *2 Standard forward voltage for single LED; *3 Standard forward current for single LED; *4 Number of LEDs used; *5 Number of series-connected LEDs; *6 Number of parallel circuits per single LED module; *7 Manufacturer: Epitex Inc., Kyoto, Japan; *8 Manufacturer: Toricon, Shimane, Japan.

**Figure 1:**
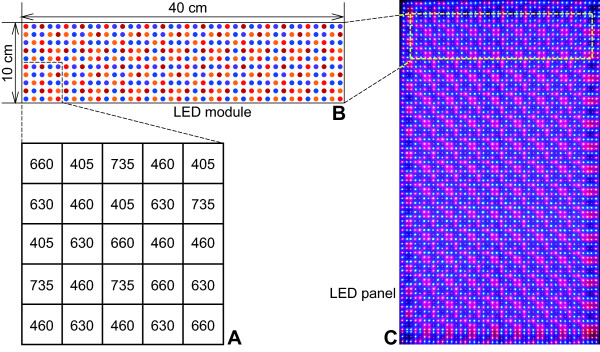
**Description of the LED panel fabricated with 2800 LEDs of five types.** The basic arrangement of 25 LEDs comprised LEDs of five types (**A**). The LED types are presented with peak wavelength (nm) shown in numerals. Repeating the basic pattern produces a 40 cm × 10 cm LED module with 400 LEDs (**B**). Seven LED modules make up the LED panel (**C**). Mirror image light spots are visible along the margin of the LED panel area in the photograph (**C**) as a result of light reflection on the glossy plates surrounding the panel (see Figure 2).

**Figure 2:**
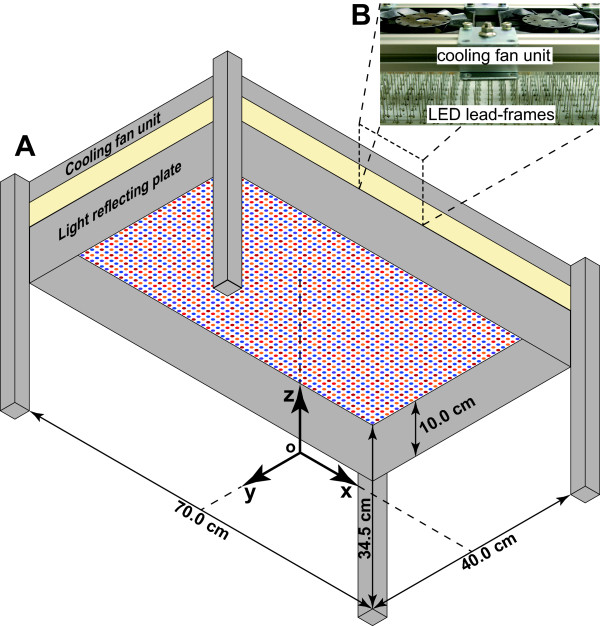
**LED panel supported with aluminum frames.** Definition of *x*-*y*-*z* coordinates (**A**) and lead-frame radiators behind the LED panel cooled by cooling fans (**B**) are presented. Origin o is on the floor below the LED panel center. The surfaces of the four plates with 10 cm width surrounding the LED panel are glossy to reflect marginal light.

Twenty-five LEDs comprised of the five types were arranged in a basic square pattern as depicted in Figure 1A. The LED module of 40 cm × 10 cm comprised 400 LEDs (Figure 1B), which were made up of 16 repeats of the basic 5 × 5 LED arrangement. The basic 5 × 5 LED arrangement on the LED module was determined such that the spectral PFD (SPFD) curves at 10 cm distance from the LED module have a similar shape for all points at the intersection of the light axes of the 25 LEDs and the irradiated surface assuming an infinitely repeated square formation of the 5 × 5 LEDs. Detailed methods of the LED arrangement determination have been described elsewhere [47]. The LED module circuit board was made of glass–epoxy, with an embedded aluminum core for efficient heat dissipation. Seven LED modules were aligned to produce a 40 cm × 70 cm LED panel (Figures 1C and 2A). To irradiate plants from the top, the LED panel was supported at 34.5 cm above the floor level using aluminum frames (Figure 2A). Four plates of 10 cm width with glossy surface enclosed the LED panel to reflect light at the panel periphery. An *xyz* coordinate system was defined as depicted in Figure 2A for representing light characteristics and plant response data according to coordinates. The lead frames of each LED were not trimmed after they were soldered to the circuit board (Figure 2B) and were left behind the circuit board to act as heat radiators. Then 28 DC fans (San Ace 80; Sanyo Electric Co. Ltd., Tokyo, Japan) were assembled above the lead-frame side of the LED panel to blow air over the lead frames. Thermal drift of irradiance emitted from the LEDs was mitigated by the cooling (details in [47]).

### PFD control system

**Figure 3:**
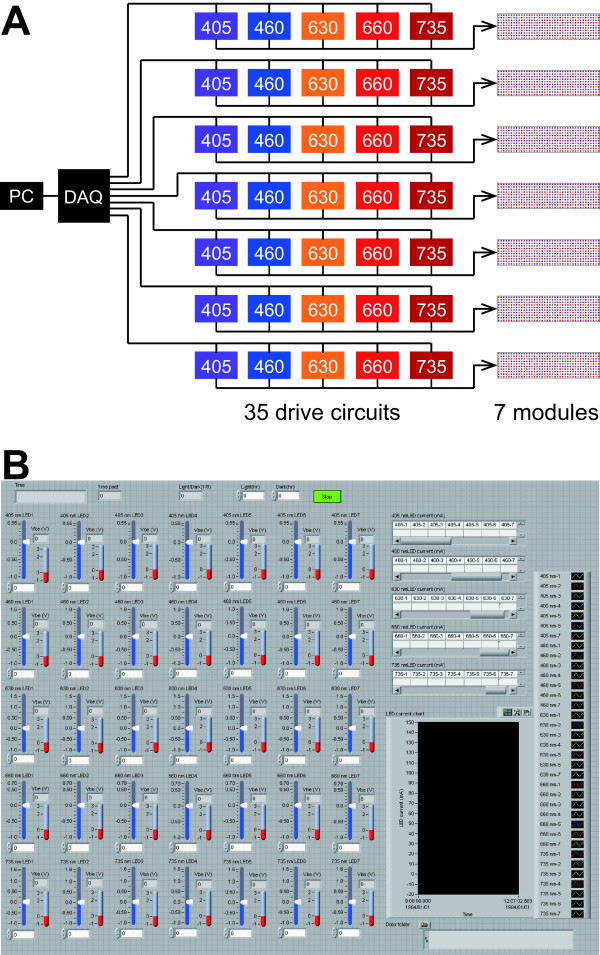
**Control system diagram of the plant lighting system and the graphical user interface.** The numerals in boxes (**A**) in the 35 drive circuits represent peak wavelengths of the five LED types. A computer (PC) controls the operation of the 35 drive circuits for LEDs of the five types and seven modules through a data acquisition system (DAQ). The graphical user interface program (**B**) enables easy operation of the 35 drive circuits merely by clicking the 35 knobs with blue indicators aligned in the 5 × 7 formation in the middle to the left of the PC screen. The 35 LED current values are fed back to the PC and are displayed on the upper right tables as numerals and on the lower right black square area as line charts (**B**).

**Figure 4:**
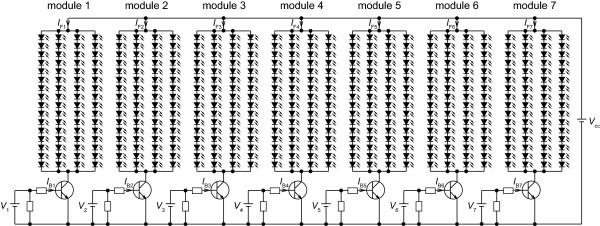
**Circuit diagram for driving the 448 violet LEDs on the seven LED modules.***V*_1_–*V*_7_ are supplied from the computer through the data acquisition system. *V*_cc_ is the power supply voltage.

**Figure 5:**
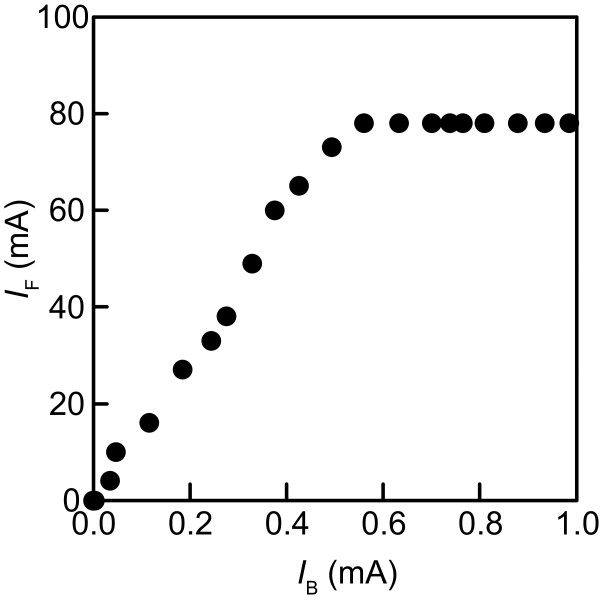
**Relation between *I***_**F**_**and *I***_**B**_**for a single violet LED drive circuit.**

Thirty-five transistor drive circuits were assembled to enable independent control of the LED forward currents *I*_F_s for regulating radiant fluxes from the five LED types and the seven LED modules (Figure 3A). The standard forward currents *I*_Fs_s for each single LED of the five types were 20 or 50 mA (Table 1). The 50 mA current was supplied to the far-red LED type. The standard forward voltages *V*_Fs_s for each single LED were 1.8–3.5 V. Shorter wavelength LEDs required higher voltage. Figure 4 presents a circuit diagram for driving of all violet LEDs on the seven LED modules. Efflux *I*_Fi_ through 64 LEDs was fed into the collector terminal of each NPN transistor (2SC4793; Toshiba Corp., Tokyo, Japan). The intensities of the LED forward currents *I*_F1_– *I*_F7_ were controlled by the respective base currents *I*_B1_– *I*_B7_ that were provided as base-emitter voltages *V*_1_– *V*_7_ from a computer through a data acquisition system (cDAQ-9172; National Instruments Corp., Texas, USA) equipped with analog voltage output modules (NI9264; National Instruments Corp., Texas, USA) (Figures 3 and 4). Voltages *V*_1_– *V*_7_ were programmed using LabVIEW software (National Instruments Corp., Texas, USA). The measured *I*_Fi_ values were fed back to the computer through the data acquisition system equipped with analog voltage input modules (NI9205; National Instruments Corp., Texas, USA). The *I*_Fi_ values were displayed on a computer monitor in numerals (shown as tables on the upper right area in Figure 3B) and line charts (shown as a black square on the lower right area in Figure 3B). A user can regulate the *I*_Fi_ values by adjusting the volume of the *V*_i_ values using the graphical user interface program (shown as 35 knobs with blue indicators in Figure 3B, the source codes are provided as Additional file 1). Figure 5 shows the relation between *I*_B_ and *I*_F_ of the single drive circuit for the violet LEDs. In fact, *I*_F_ increased linearly until 80 mA, which is the upper limit value for the four-parallel LED circuits on the single LED module. The upper limit was controlled by the function of the power supply *V*_cc_ (PAS80-4.5; Kikusui Electronics Corp., Yokohama, Japan). The single power supply fed electric power to all LEDs of the same type on the LED panel. Therefore, five power supplies were used for driving LEDs of the five types.

### Light measurements

Below the LED panel at *z* = 17.3 cm, SPFDs were measured at 91 points across *x* = ±30 cm and *y* = ±15 cm at 5 cm intervals for wavelengths of 350–800 nm using a spectroradiometer (MS-720; Eko Instruments Co., Ltd., Tokyo, Japan). Although the LED panel irradiated 40 cm × 70 cm area, the marginal 5 cm of the irradiated area was excluded from light measurements because light sharply changes such a marginal area and plants are unlikely to be positioned there. The position *z* = 17.3 cm corresponds with the height of the spectroradiometer sensor position from its bottom, which was placed on the floor. Independent PFDs emitted by each LED type alone were estimated from the measured single SPFD curve with five peaks when *I*_Fs_s were fed to every LED. A Gaussian function was used to separate the five independent PFDs from the measured SPFD curve. The optimum Gaussian function was ascertained using Mathematica 7 (Wolfram Research, Inc., Illinois, USA) with a coefficient of determination *r*^2^ of greater than 0.97.

### Plant irradiation using the lighting system

To assay plant sensitivities to the light control quality of the plant lighting system, the phototropic response of oat coleoptiles to an SPFD gradient of blue light was investigated. Oat seeds (*Avena sativa* L. cv. Super-hayate) were purchased from Snow Brand Seed Co. Ltd. (Sapporo, Japan). The plant lighting system was placed in a dark room where the room air temperature was maintained at 24.5 ± 1.5°C. A cell tray with 2.5 cm cell intervals was positioned below the LED panel. Vermiculite was put in each cell so that the vermiculite surface was positioned at *z* = 17 cm. The vermiculite was watered with tap water before sowing. Thirty-six oat seeds were sown in the tray cells as one seed per single cell. The 36 seeds were positioned at *x* = ±1.3, ±3.8, ±6.3, ±8.8, ±11.3, and ±13.8 cm at *y* = 0 and ±2.5 cm. After 66 h, the LEDs started to irradiate the seeds with the SPFD value of 1 μmol m^–2^ s^–1^ nm^–1^ at all five *λ*_p_s at *z* = 17.3 cm. After 23 h irradiation, every germinated seedling was photographed using a digital camera (E-P1; Olympus Corp., Tokyo, Japan) from the -*y* direction. The phototropic curvature of coleoptiles was measured from digital images as an angle formed by the *z* axis and central axis of each coleoptile. No-bending vertical growth of coleoptiles was defined as 0 deg curvature. The curvature of coleoptiles to the positive *x* directions was defined as the positive curvature. This series of experiments was repeated four times. The next series of experiments was conducted five times similarly to the description presented above, but only the blue light (*λ*_p_ = 460 nm) SPFD had a gradient in the *x* direction by gradually differentiating the *I*_F_ values of each module’s blue LEDs. The SPFD gradient was defined as the change of the SPFD value (μmol m^–2^ s^–1^ nm^–1^) per displacement (m) along the *x* direction. Therefore, the SPFD gradient unit becomes μmol m^–2^ s^–1^ nm^–1^ m^–1^. Consequently, the unit which represents the relation between the phototropic curvature (deg) and the blue-light SPFD gradient (μmol m^–2^ s^–1^ nm^–1^ m^–1^) becomes deg per μmol m^–2^ s^–1^ nm^–1^ m^–1^. The blue light spectrum with a peak at around 460 nm wavelength is known to induce phototropic responses in higher plants [23,37,48].

## Results

### PFD and mixing ratio control

**Figure 6:**
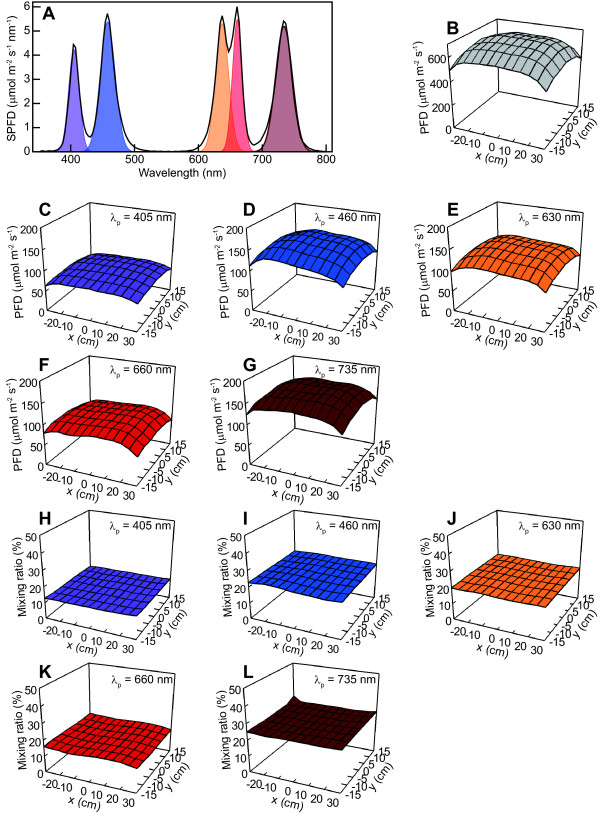
**SPFD, PFD, and mixing ratio of PFD for each LED type.** Independent five SPFDs (**A**, colored) emitted by each LED type alone were estimated from the measured single SPFD curve (**A**, solid black line) at *x* = *y* = 0 at *z* = 17.3 cm, when standard forward current *I*_Fs_s were fed to every LED. Gaussian function was used to separate the five peaks from the measured SPFD curve (**A**). Distributions of PFDs (**C**–**G**) at the irradiated area (*x* = ±30 cm and *y* = ±15 cm at *z* = 17.3 cm) provided from each LED type alone were determined using Gaussian function with the estimation error of less than 3.71% for the 91 points of the irradiated area. The mixing ratios of each LED type’s PFD against the total PFD (**B**) were calculated for the irradiated area (**H**–**L**).

The average PPFD (coefficient of variation (CV)) of the 91 measurement points in the irradiated area (*x* = ±30 cm and *y* = ±15 cm at *z* = 17.3 cm) was 438 μmol m^–2^ s^–1^ (9.6%) when the *I*_Fs_s were supplied to all LEDs. SPFD values at the five *λ*_p_s deviated between 4.4 μmol m^–2^ s^–1^ nm^–1^ at *λ*_p_ = 405 nm to 6.0 μmol m^–2^ s^–1^ nm^–1^ at *λ*_p_ = 660 nm (Figure 6A, black solid line). A Gaussian function separated five respective PFDs (Figure 6A, colored peaks) from the measured SPFD curve (Figure 6A, black solid line) with the PFD estimation errors of less than 3.71% for the 91 irradiated points (*x* = ±30 cm, *y* = ±15 cm, and *z* = 17.3 cm). The average PFDs (μmol m^–2^ s^–1^) (CV) of the irradiated area for the total and each LED type alone were, respectively, 610.3 (9.3%) for the total, 77.7 (9.4%) for violet, 139.9 (10.0%) for blue, 123.5 (9.5%) for orange-red, 94.4 (9.6%) for red, and 153.9 (8.6%) for far-red. The mixing ratios of each LED type’s PFD (Figure 6C–6G) against the total PFD (Figure 6B) were calculated for the 91 points. They are depicted as Figure 6H–6L. The mixing ratios among the LED types differed when *I*_Fs_s were provided, but the mixing ratio distributions of each LED type in the *x*-*y* plane were uniform.

**Figure 7:**
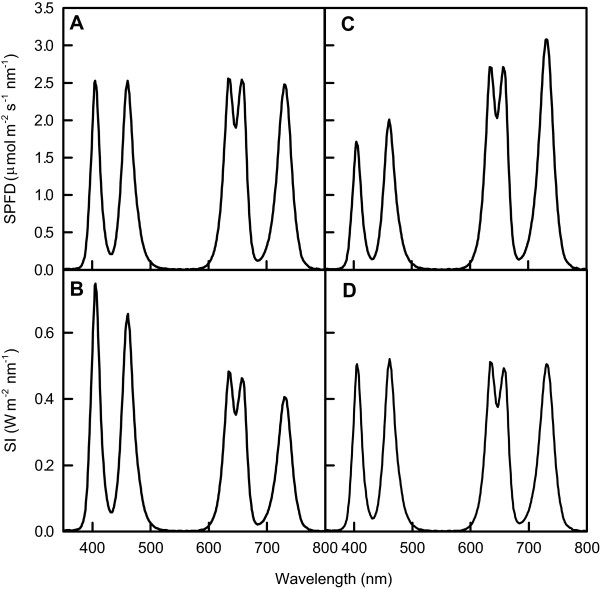
**Mixing ratio controlled SPFD and SI values at the five *λ***_**p**_**s.** The SPFD values were adjusted at 2.5 μmol m^–2^ s^–1^ nm^–1^ (**A**) with the corresponding SI curve (**B**). The SI values were adjusted to 0.5 W m^–2^ nm^-1^ (**D**) with the corresponding SPFD curve (**C**). The SPFD and SI values were measured at *x* = *y* = 0 and *z* = 17.3 cm.

Some plant light response studies require a unique mixing ratio of light spectrum peaks. As a demonstration of mixing ratio controllability, all SPFD values at the five *λ*_p_s were adjusted to the same value by regulation of the *I*_F_ values of the five type LEDs (Figure 7A). In this case, the spectral irradiance (SI) (W m^–2^ nm^–1^) values at the shorter *λ*_p_s were greater than those at the longer *λ*_p_s (Figure 7B) because photons with shorter wavelengths have greater energy. This SPFD controllability is expected to be valid for photon-number-based studies such as those of photosynthesis [6]. Alternatively, SI values at the five *λ*_p_s could be adjusted to the same value (Figure 7D). In such a case, SPFD values at the longer *λ*_p_s were greater than those at the shorter *λ*_p_s (Figure 7C) because the number of photons with longer wavelengths must be greater than that of photons with shorter wavelengths to emit the same energy level light. This SI value controllability is an important feature when the lighting quantity is assessed on an energy basis [6].

**Figure 8:**
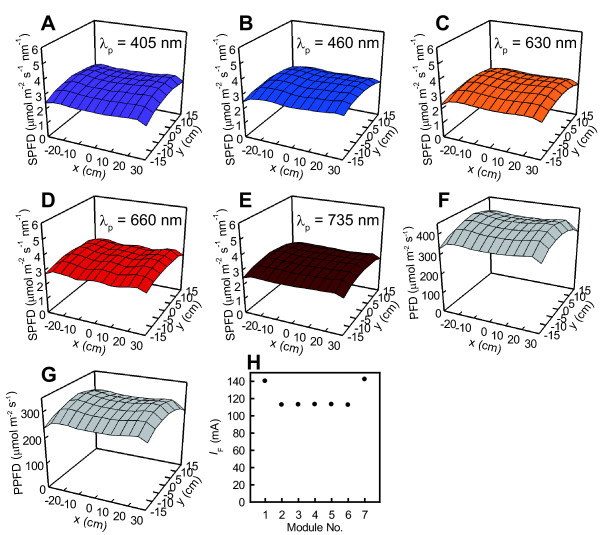
**Distributions of SPFDs, PFD, and PPFD and forward current *I***_**F**_**s for the far-red LED type.** Uniformly controlled SPFD distributions at 3 μmol m^–2^ s^–1^ nm^–1^ within the area of *x* = ±30 cm and *y* = ±15 cm at *z* = 17.3 cm at *λ*_p_ = 405 nm (**A**), 460 nm (**B**), 630 nm (**C**), 660 nm (**D**), and 735 nm (**E**) are depicted. Distribution of PFD (**F**) and PPFD (**G**) when the lighting system irradiated the uniform SPFD distribution (**A**–**E**) are also presented. The PFD declination along the *x* direction was improved by supplying greater *I*_F_ values to the outermost modules’ LEDs (**H**).

Although the LED panel was enclosed within the glossy plates to reflect rim light to the inside, the PFD declined at the peripheries of the irradiated area. However, this decline along the *x* direction was improved by supplying greater *I*_F_ values to the outermost modules’ LEDs (Figure 8H). No controllability was available for improving the PFD decline in the *y* direction, but the PFDs were fairly uniform across the *y* direction. An example of uniformly distributed SPFD at 3 μmol m^–2^ s^–1^ nm^–1^ at every *λ*_p_ in the area of *x* = ±30 cm and *y* = ±15 cm at z = 17.3 cm is depicted in Figure 8 (A–E). Averages of the 91 SPFD (μmol m^–2^ s^–1^ nm^–1^) (CV) within the area were 2.9 (8.1%) at *λ*_p_ = 405 nm, 3.0 (8.1%) at *λ*_p_ = 460 nm, 2.9 (9.0%) at *λ*_p_ = 630 nm, 3.1 (6.6%) at *λ*_p_ = 660 nm, and 2.9 (7.2%) at *λ*_p_ = 735 nm. The averages of the 91 PFD (μmol m^–2^ s^–1^) (CV) and PPFD (μmol m^–2^ s^–1^) (CV) within the area were, respectively 392 (7.5%) and 286 (7.6%) (Figure 8F and 8G) when the lighting system irradiated the 3 μmol m^–2^ s^–1^ nm^–1^ SPFD distribution.

### Phototropic curvature of oat coleoptiles

**Figure 9:**
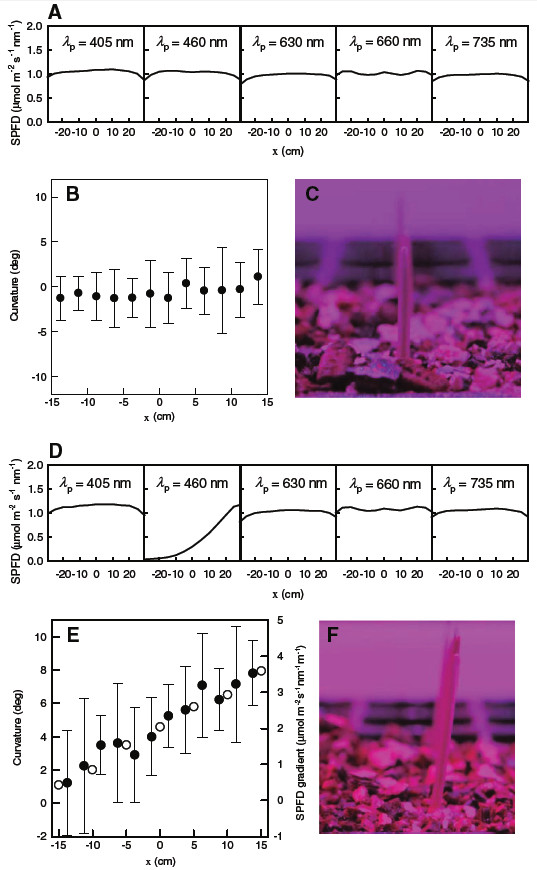
**Oat coleoptile curvatures under uniform or blue-gradient SPFD irradiation.** Uniform distribution of SPFD at *λ*_p_ = 405, 460, 630, 660, and 735 nm along the *x*-axis at *y* = 0 and *z* = 17.3 cm (**A**), curvature of oat coleoptiles (**B**) when irradiated for 23 h with the SPFD depicted in (**A**), and a photograph of vertically growing coleoptiles (**C**) at *x* = −11.3 cm are presented. Modified distribution of SPFD at *λ*_p_ = 460 nm (**D**), curvature of oat coleoptiles (solid circles in **E**) when irradiated with the SPFD depicted in (**D)**, and a photograph of coleoptiles showing positive curvature (**F**) at *x* = +11.3 cm are presented. The SPFD gradient at *λ*_p_ = 460 nm along the *x*-axis at *y* = 0 and *z* = 17.3 cm is depicted as open circles in (**E**). No-bending vertical coleoptile growth is defined as 0 deg curvature. Coleoptile curvature to positive *x* is defined as positive curvature. Bars in (**B**) and (**E**) represent standard deviations.

Oat coleoptiles were irradiated for 23 h with the uniformly distributed 1 μmol m^–2^ s^–1^ nm^–1^ SPFD light at every *λ*_p_, as depicted in Figure 9A. The coleoptiles grew almost straight upwards at all *x* positions (Figure 9B and 9C), where three coleoptiles per single *x* position (i.e., *y* = 0 and ±2.5 cm at each *x* position) were examined with the same population sample because the SPFD deviation across the *y* direction around *y* = 0 was negligible (Figure 8). The number of plant samples at each *x* position was 7–13 because non-germinated seeds were not counted in the sample. The oat coleoptiles irradiated with blue (*λ*_p_ = 460 nm) gradient light of which the SPFD had been decreased from 1 μmol m^–2^ s^–1^ nm^–1^ at *x* = 20 cm to 0 μmol m^–2^ s^–1^ nm^–1^ at *x* = −20 cm (Figure 9D) bent to the positive *x* direction (Figure 9E and 9F). The greater SPFD gradient induced greater curvature of coleoptiles (Figure 9E). The relation between the phototropic curvature and the blue-light SPFD gradient was 2 deg per 1 μmol m^–2^ s^–1^ nm^–1^ m^–1^.

## Discussion

Light is among the most difficult physical environmental factors to regulate uniformly in terms of its distribution and temporal control of intensity throughout plant experiments. For field or greenhouse experiments, temporal consistency of natural light is impossible to achieve. In a growth chamber experiment, uniform distribution of light is difficult to achieve because of the chamber limitation and light source size. As this experiment demonstrated, using numerous concentrated small lighting elements enables more uniform distribution of lighting than that obtained using a few large light sources such as incandescent and fluorescent lamps. This advantage ensures uniform plant response when many seedlings are grown below the LED panel. The lighting system produced average PPFD of 438 μmol m^–2^ s^–1^ at *z* = 17.3 cm, where plants are likely to be positioned. This value was sufficiently high to grow leafy vegetables [49]. PFD values are continuously controllable from zero to the maximum value by regulating electric current fed to respective LED types. Transistor circuits with a computer signal control were effective for this purpose (Figures 4 and 5). The computer program with the graphical user interface assists manifold variations of PFD and mixing ratio designs for plant irradiation. This usability enables the lighting system to emit either a fairly even distribution (Figures 8 and 9A) or an intentionally distorted distribution (Figure 9D) of light to a plant canopy below the LED panel through control of PFD mixing ratio.

Results of the phototropism experiments suggest the importance of attentive adjustment of light characteristics in a plant experiment environment. Uniform lighting can induce uniform and reproducible plant responses, thereby delivering increased stringency for investigation of complex plant functions. The mixing ratio controllability of PFDs also enables us to study plant light responses to inhomogeneous light distribution. This study demonstrated a linear relationship, under our experimental conditions, between a blue-light SPFD gradient and the oat coleoptile curvature (Figure 9E). The PFD controllability of this lighting system is not limited to blue light. For example, a mixing ratio gradient of red/far-red, which often occurs in natural and cultivation environments, has attracted the attention of plant scientists [40,43,44]. The present lighting system is suitable for such studies as well.

Although the present lighting system provides five peak wavelengths, an apparent limitation is the wavelength coverage of emitted light. Physiologically important green light [50-55] should be included in future versions of plant lighting systems. Furthermore, as plant light response studies advance, light of more varied spectra will be anticipated for emission by artificial lighting systems. Plants have evolved under sunlight. For that reason, they may use a full range of the ground level sunlight spectrum. Hogewoning et al. [56] reported striking growth enhancement of cucumber plants irradiated with an artificial quasi-solar-spectrum light compared to cucumber growth when irradiated with fluorescent or high-pressure sodium lamps. In principle, quasi-ground-level-sunlight spectra are producible using various combinations of LEDs [57-59]. The LED lighting systems are expected to contribute substantially to a better understanding of the nature of plant light responses.

## Conclusions

For control of both the PFD and mixing ratio of illumination, we developed a five-wavelength-band plant lighting system using 2800 LEDs of five types. The SPFD values were controlled uniformly at the irradiated area of 30 cm × 60 cm. Alternatively, an intentionally distorted SPFD gradient could be created. A computer graphical user interface facilitated the adjustment of these lighting parameters. The SPFD control performance was tested through the phototropic response of oat coleoptiles. The oat coleoptiles grew straight upward under a uniform SPFD distribution below the LED panel. On the other hand, phototropic curvature was induced by a blue light (*λ*_p_ = 460 nm) gradient, suggesting the merit of PFD and the mixing ratio controllability of the LED plant lighting system.

## Competing interests

The authors declare that they have no competing interests.

## Authors’ contributions

AY designed the LED drive circuits and cooling unit, programmed the LabVIEW interface software, conducted light measurements and phototropism experiments, and drafted the manuscript. KF organized the LED system development, designed the LED arrangements and the lighting unit structure, and critically reviewed the manuscript. All authors read and approved the final manuscript.

## Supplementary Material

Additional file 1 **Source codes file.** This program was produced using LabVIEW 2010 (National Instruments Corp., Texas, USA). The 35 drive circuit operations for the five LED types and the seven modules were controlled by these source codes (mainly 5 × 7 pale blue icons). LED current values of the 35 drive circuits are fed back to the computer, are stored in a file, and are displayed on the computer screen as tables and charts (bottom left area with five pink icons). Light and dark periods are controllable (top left area with orange connection lines). Click here for file
