# Supplementary figures and images for: Plant lighting system with five wavelength-band light-emitting diodes providing photon flux density and mixing ratio control
Source: Plant Methods. 2012 Nov 22;8:46. doi: 10.1186/1746-4811-8-46 (PMC3573946; doi:10.1186/1746-4811-8-46)

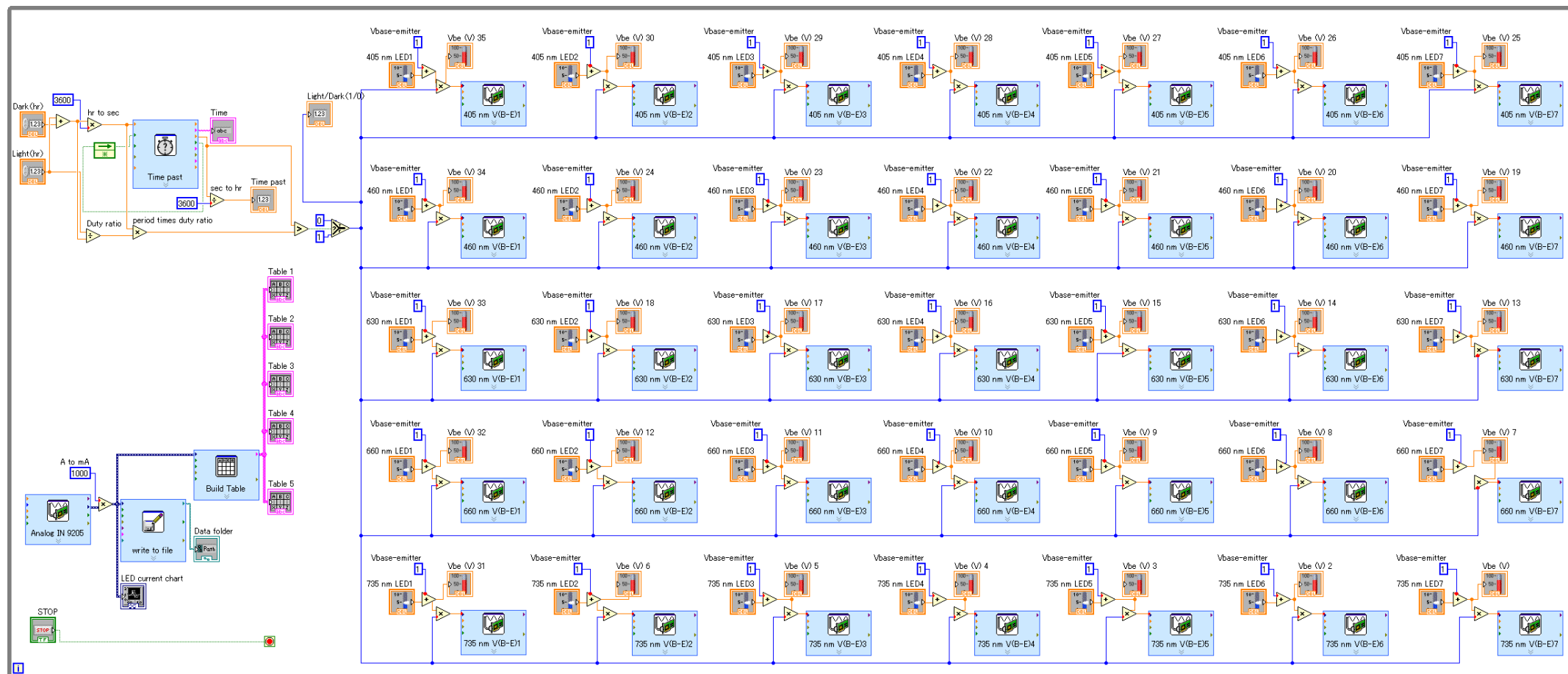

Supplement: Additional file 1 — Source codes file. This program was produced using LabVIEW 2010 (National Instruments Corp., Texas, USA). The 35 drive circuit operations for the five LED types and the seven modules were controlled by these source codes (mainly 5 × 7 pale blue icons). LED current values of the 35 drive circuits are fed back to the computer, are stored in a file, and are displayed on the computer screen as tables and charts (bottom left area with five pink icons). Light and dark periods are controllable (top left area with orange connection lines). [file 1746-4811-8-46-S1.pdf]
